# Supplementary material for: Single Nucleotide Polymorphisms as Biomarkers of Response to Neoadjuvant Chemoradiotherapy in Rectal Cancer: A Systematic Review
Source: Cancers (Basel). 2025 Dec 15;17(24):3995. doi: 10.3390/cancers17243995 (PMC12730669; doi:10.3390/cancers17243995)
Supplement: Supplementary file 1 [file cancers-17-03995-s001.zip › cancers-4010303-supplementary.docx]

*Systematic Review*

**Single Nucleotide Polymorphisms as Biomarkers of Response to Neoadjuvant Chemoradiotherapy in Rectal Cancer:
A Systematic Review**

**Supplement S1. Summary of SNPs not related to treatment response in at least one study.**

| **SNP** | **Gene** | **Papers** | **Number of studies** |
| --- | --- | --- | --- |
| rs1045642 | ABCB1 | Cecchin,Formica,Kim | 3 |
| rs1128503 | ABCB1 | Cecchin,Kim | 2 |
| rs2032582 | ABCB1 | Kim | 1 |
| rs2273697 | ABCC2 | Cecchin | 1 |
| rs717620 | ABCC2 | Cecchin | 1 |
| rs1136410 | ADPRT | Ho-Pun-Cheung | 1 |
| rs1130214 | AKT1 | Peng | 1 |
| rs2498804 | AKT1 | Peng | 1 |
| rs2494738 | AKT1 | Peng | 1 |
| rs8100018 | AKT2 | Peng | 1 |
| rs1130409 | APEX1 | Dreussi,Ho-Pun-Cheung | 2 |
| rs1760944 | APEX1 | Ho-Pun-Cheung | 1 |
| rs11942466 | AREG | Sebio | 1 |
| rs1353295 | AREG | Sebio | 1 |
| rs10034692 | AREG | Sebio | 1 |
| rs6447003 | AREG | Sebio | 1 |
| rs2132065 | AREG | Sebio | 1 |
| rs11725706 | AREG | Sebio | 1 |
| rs3913032 | AREG | Sebio | 1 |
| rs13104811 | AREG | Sebio | 1 |
| rs28635876 | AREG | Sebio | 1 |
| rs7808424 | ASZ1 | Kim | 1 |
| rs1801516 | ATM | Dreussi,Ho-Pun-Cheung | 2 |
| rs189037 | ATM | Ho-Pun-Cheung | 1 |
| rs1800057 | ATM | Ho-Pun-Cheung | 1 |
| rs36017265 | BAX | Ho-Pun-Cheung | 1 |
| rs4645878 | BAX | Ho-Pun-Cheung | 1 |
| rs2279115 | BCL2 | Ho-Pun-Cheung | 1 |
| rs1799966 | BRCA1 | Ho-Pun-Cheung | 1 |
| rs16942 | BRCA1 | Ho-Pun-Cheung | 1 |
| rs799917 | BRCA1 | Ho-Pun-Cheung | 1 |
| rs16941 | BRCA1 | Ho-Pun-Cheung | 1 |
| rs1799943 | BRCA2 | Ho-Pun-Cheung | 1 |
| rs206143 | BRCA2 | Ho-Pun-Cheung | 1 |
| rs144848 | BRCA2 | Ho-Pun-Cheung | 1 |
| rs6948 | CASP3 | Ho-Pun-Cheung | 1 |
| rs1049216 | CASP3 | Ho-Pun-Cheung | 1 |
| rs1045485 | CASP8 | Ho-Pun-Cheung | 1 |
| rs13113 | CASP8 | Ho-Pun-Cheung | 1 |
| rs1052576 | CASP9 | Ho-Pun-Cheung | 1 |
| rs13010627 | CASP10 | Ho-Pun-Cheung | 1 |
| rs1001179 | CAT | Leu | 1 |
| rs769214 | CAT | Leu | 1 |
| rs603965 | CCND1 | Garcia-Aguilar,Ho-Pun-Cheung,Ho-Pun-Cheung | 3 |
| rs9344 | CCND1 | Hu-Lieskovan | 1 |
| rs192986 | CDC42BPA | Kim | 1 |
| rs1801270 | CDKN1A | Ho-Pun-Cheung | 1 |
| rs521102 | CHEK1 | Ho-Pun-Cheung | 1 |
| rs2267130 | CHEK2 | Ho-Pun-Cheung | 1 |
| rs11772832 | CNOT4 | Dreussi | 1 |
| rs6877400 | CNOT6 | Dreussi | 1 |
| rs1985859 | CORO2A | Kim | 1 |
| rs4135385 | CTNNB1 | Ho-Pun-Cheung | 1 |
| rs13072632 | CTNNB1 | Ho-Pun-Cheung | 1 |
| rs1049255 | CYBA | Leu | 1 |
| rs1048943 | CYP1A1 | Ho-Pun-Cheung | 1 |
| rs5031016 | CYP2A6 | Kim | 1 |
| rs28399468 | CYP2A6 | Kim | 1 |
| rs28399433 | CYP2A6 | Kim | 1 |
| rs28399468 | CYP2A6 | Kim | 1 |
| rs197412 | DDX20 | Dreussi | 1 |
| rs417309 | DGCR8 | Dreussi | 1 |
| rs1057035 | DICER1 | Dreussi | 1 |
| rs3918290 | DPYD | Balboa | 1 |
| rs10719 | DROSHA | Dreussi | 1 |
| rs4444903 | EGF | Dreussi,Ho-Pun-Cheung,Hu-Lieskovan,Sebio | 4 |
| rs6533485 | EGF | Sebio | 1 |
| rs11568993 | EGF | Sebio | 1 |
| rs4698803 | EGF | Sebio | 1 |
| rs11568972 | EGF | Sebio | 1 |
| rs929446 | EGF | Sebio | 1 |
| rs2074390 | EGF | Sebio | 1 |
| rs6850557 | EGF | Sebio | 1 |
| rs11568315 | EGFR | Balboa | 1 |
| rs2227983 | EGFR | Dreussi,Hu-Lieskovan,Paez,Sebio | 4 |
| rs17290169 | EGFR | Ho-Pun-Cheung | 1 |
| rs17335738 | EGFR | Ho-Pun-Cheung | 1 |
| rs712830 | EGFR | Ho-Pun-Cheung,Sebio | 2 |
| rs712829 | EGFR | Ho-Pun-Cheung,Sebio,Spindler | 3 |
| rs11543848 | EGFR | Ho-Pun-Cheung | 1 |
| rs1801200 | ERBB2 | Ho-Pun-Cheung | 1 |
| rs11615 | ERCC1 | Balboa,Cecchin,Dreussi,Formica,Ho-Pun-Cheung,Lamas,Paez,Sebio | 8 |
| rs10412761 | ERCC1 | Boige | 1 |
| rs3212986 | ERCC1 | Boige,Cecchin,Dreussi,Paez,Sebio | 5 |
| rs2298881 | ERCC1 | Boige | 1 |
| rs2336219 | ERCC1 | Boige | 1 |
| rs4803823 | ERCC1 |  |  |
| rs3212948 | ERCC1 | Paez | 1 |
| rs13181 | ERCC2 (XPD) | Balboa,Boige,Cecchin,Paez,Sebio | 5 |
| rs50871 | ERCC2 (XPD) | Boige | 1 |
| rs11878644 | ERCC2 (XPD) | Boige | 1 |
| rs50872 | ERCC2 (XPD) | Boige | 1 |
| rs238415 | ERCC2 (XPD) | Boige | 1 |
| rs1799793 | ERCC2 (XPD) | Boige,Cecchin,Dreussi | 3 |
| rs238415 | ERCC2 (XPD) | Boige | 1 |
| rs28365048 | ERCC2 (XPD) | Ho-Pun-Cheung | 1 |
| rs1799787 | ERCC2 (XPD) | Boige | 1 |
| rs1364362 | ERCC4 | Boige | 1 |
| rs1800067 | ERCC4 | Boige | 1 |
| rs11075223 | ERCC4 | Boige | 1 |
| rs1799802 | ERCC4 | Ho-Pun-Cheung | 1 |
| rs744154 | ERCC4 | Ho-Pun-Cheung | 1 |
| rs1799801 | ERCC4 | Ho-Pun-Cheung | 1 |
| rs1799800 | ERCC4 | Ho-Pun-Cheung | 1 |
| rs17655 | ERCC5 | Ho-Pun-Cheung | 1 |
| rs7687621 | EREG | Sebio | 1 |
| rs1017733 | EREG | Sebio | 1 |
| rs4149963 | EXO1 | Dreussi | 1 |
| rs7955740 | FAM101A | Kim | 1 |
| rs1801274 | FCGR2A | Ho-Pun-Cheung,Hu-Lieskovan | 2 |
| rs396991 | FCGR3A | Ho-Pun-Cheung,Hu-Lieskovan | 2 |
| rs308447 | FGF2 | Ho-Pun-Cheung | 1 |
| rs351855 | FGFR4 | Ho-Pun-Cheung | 1 |
| rs867228 | FPR1 | Chiang | 1 |
| rs2295080 | FRAP1 | Peng | 1 |
| rs11121704 | FRAP1 | Peng | 1 |
| rs1050450 | GPX1 | Ho-Pun-Cheung,Leu | 2 |
| rs334558 | GSK3B | Ho-Pun-Cheung | 1 |
| rs3755557 | GSK3B | Ho-Pun-Cheung | 1 |
| rs3957357 | GSTA1*B | Cecchin | 1 |
| rs7927381 | GSTP1 | Boige | 1 |
| rs1138272 | GSTP1 | Boige | 1 |
| rs6591256 | GSTP1 | Boige | 1 |
| rs947894 | GSTP1 | Cecchin | 1 |
| rs1138272 | GSTP1 | Cecchin,Dreussi | 2 |
| rs1695 | GSTP1 | Dreussi,Formica,Ho-Pun-Cheung,Nicosia,Paez | 5 |
| rs4630 | GSTT1 | Ho-Pun-Cheung | 1 |
| rs11549465 | HIF1A | Havelund,Ho-Pun-Cheung | 2 |
| rs11549467 | HIF1A | Havelund,Ho-Pun-Cheung | 2 |
| rs2057482 | HIF1A | Havelund | 1 |
| rs2246350 | HIF1A | Ho-Pun-Cheung | 1 |
| rs1056538 | ICAM5 | Ho-Pun-Cheung | 1 |
| rs2228615 | ICAM5 | Ho-Pun-Cheung | 1 |
| rs2229765 | IGF1 | Ho-Pun-Cheung | 1 |
| rs629849 | IGF2R | Ho-Pun-Cheung | 1 |
| rs16944 | IL1B | Dzhugashvili | 1 |
| rs1143627 | IL1B | Dzhugashvili,Ho-Pun-Cheung | 2 |
| rs1143634 | IL1B | Ho-Pun-Cheung | 1 |
| rs2243250 | IL4 | Ho-Pun-Cheung | 1 |
| rs1800795 | IL6 | Ho-Pun-Cheung | 1 |
| rs4073 | IL8 | Ho-Pun-Cheung,Hu-Lieskovan | 2 |
| rs1800896 | IL10 | Ho-Pun-Cheung | 1 |
| rs20541 | IL13 | Ho-Pun-Cheung | 1 |
| rs1800925 | IL13 | Ho-Pun-Cheung,Xiao | 2 |
| rs61764370 | KRAS | Hu-Lieskovan,Sclafani | 2 |
| rs1052536 | LIG3 | Ho-Pun-Cheung | 1 |
| rs3135967 | LIG3 | Ho-Pun-Cheung | 1 |
| rs1805388 | LIG4 | Ho-Pun-Cheung | 1 |
| rs1805386 | LIG4 | Ho-Pun-Cheung | 1 |
| rs2229094 | LTA | Ho-Pun-Cheung | 1 |
| rs10342 | MBD4 | Ho-Pun-Cheung | 1 |
| rs140693 | MBD4 | Ho-Pun-Cheung | 1 |
| rs2279744 | MDM2 | Dreussi | 1 |
| rs1470383 | MDM2 | Ho-Pun-Cheung | 1 |
| rs1571256 | MED4 | Kim | 1 |
| rs12917 | MGMT | Dreussi,Ho-Pun-Cheung | 2 |
| rs4919510 | miR-608 | Sclafani | 1 |
| rs11614913 | miR196A2 | Dreussi | 1 |
| rs28461391 | miR371A | Dreussi | 1 |
| rs1799977 | MLH1 | Cecchin,Dreussi,Ho-Pun-Cheung | 3 |
| rs1800734 | MLH1 | Ho-Pun-Cheung | 1 |
| rs7208693 | MPO | Ho-Pun-Cheung | 1 |
| rs2333227 | MPO | Leu | 1 |
| rs2303428 | MSH2 | Cecchin | 1 |
| rs3136228 | MSH6 | Dreussi | 1 |
| rs2853826 | MT-ND3 | Ho-Pun-Cheung | 1 |
| rs3737967 | MTHFR | Boige | 1 |
| rs3818762 | MTHFR | Boige | 1 |
| rs3737964 | MTHFR | Boige | 1 |
| rs7553194 | MTHFR | Boige | 1 |
| rs17367504 | MTHFR | Boige | 1 |
| rs9651118 | MTHFR | Boige | 1 |
| rs4846052 | MTHFR | Boige | 1 |
| rs1572151 | MTHFR | Boige | 1 |
| rs1801133 | MTHFR | Boige,Cecchin,Dreussi,Garcia-Aguilar,Ho-Pun-Cheung,Hu-Lieskovan,Lamas,Nikas,Stanojevic,Terrazzino | 10 |
| rs1801131 | MTHFR | Boige,Cecchin,Dreussi,Ho-Pun-Cheung,Hu-Lieskovan,Lamas,Balboa,Formica,Stanojevic,Terrazzino | 10 |
| rs17375901 | MTHFR | Boige | 1 |
| rs1805794 | NBN | Ho-Pun-Cheung | 1 |
| rs5031039 | NFE2L2 | Ho-Pun-Cheung | 1 |
| 686A>G | NFE2L2 | Ho-Pun-Cheung | 1 |
| 650C>A | NFE2L2 | Ho-Pun-Cheung | 1 |
| rs3774932 | NFKB1 | Ho-Pun-Cheung | 1 |
| rs3774937 | NFKB1 | Ho-Pun-Cheung | 1 |
| rs3774934 | NFKB1 | Ho-Pun-Cheung | 1 |
| rs3774936 | NFKB1 | Ho-Pun-Cheung | 1 |
| rs2297518 | NOS2A | Ho-Pun-Cheung | 1 |
| rs179998 | NOS3 | Ho-Pun-Cheung | 1 |
| rs1052133 | OGG1 | Cecchin,Dreussi,Ho-Pun-Cheung,Leu | 4 |
| rs1538704 | OR2T4 | Kim | 1 |
| rs3751143 | P2RX7 | Chiang | 1 |
| rs11136410 | PARP-1 | Dreussi,Ho-Pun-Cheung | 2 |
| rs2699887 | PIK3CA | Peng | 1 |
| rs6443624 | PIK3CA | Peng | 1 |
| rs7621329 | PIK3CA | Peng | 1 |
| rs7651265 | PIK3CA | Peng | 1 |
| rs1801282 | PPARG | Ho-Pun-Cheung | 1 |
| rs2299939 | PTEN | Peng | 1 |
| rs12569998 | PTEN | Peng | 1 |
| rs1213266 | PTGS1 | Dzhugashvili | 1 |
| rs5789 | PTGS1 | Dzhugashvili | 1 |
| rs5275 | PTGS2 | Dzhugashvili | 1 |
| rs20417 | PTGS2 | Ho-Pun-Cheung,Hu-Lieskovan | 2 |
| rs1801320 | RAD51 | Cecchin,Hu-Lieskovan | 2 |
| rs5030783 | RAD51 | Ho-Pun-Cheung | 1 |
| rs1801321 | RAD51 | Ho-Pun-Cheung | 1 |
| rs11226 | RAD52 | Ho-Pun-Cheung | 1 |
| rs13035 | RECQL | Ho-Pun-Cheung | 1 |
| rs41398848 | SLC10A7 | Kim | 1 |
| rs1792671 | SMAD2 | Dreussi | 1 |
| rs17228212 | SMAD3 | Dreussi | 1 |
| rs744910 | SMAD3 | Dreussi | 1 |
| rs8028147 | SMAD3 | Dreussi | 1 |
| rs8025774 | SMAD3 | Dreussi | 1 |
| rs745103 | SMAD3 | Dreussi | 1 |
| rs2289791 | SMAD3 | Dreussi | 1 |
| rs1057898 | SMAD5 | Dreussi | 1 |
| rs6871224 | SMAD5 | Dreussi | 1 |
| rs4880 | SOD2 | Boige | 1 |
| rs5746136 | SOD2 | Boige | 1 |
| rs5746141 | SOD2 | Boige | 1 |
| rs2842980 | SOD2 | Boige | 1 |
| rs2758329 | SOD2 | Boige | 1 |
| rs4342445 | SOD2 | Boige | 1 |
| rs4880 | SOD2 | Dreussi,Leu,Ho-Pun-Cheung | 3 |
| rs699473 | SOD3 | Leu | 1 |
| rs2736108 | TERT | Rampazzo | 1 |
| rs2853690 | TERT | Rampazzo | 1 |
| rs35241335 | TERT | Rampazzo | 1 |
| rs2736100 | TERT | Rampazzo | 1 |
| rs2736098 | TERT | Rampazzo | 1 |
| rs11742908 | TERT | Rampazzo | 1 |
| rs2736122 | TERT | Rampazzo | 1 |
| rs2735940 | TERT | Rampazzo | 1 |
| rs1982073 | TGFB1 | Ho-Pun-Cheung | 1 |
| rs1800469 | TGFB1 | Ho-Pun-Cheung | 1 |
| rs1800471 | TGFB1 | Ho-Pun-Cheung | 1 |
| rs1036199 | TIM3 | Chiang | 1 |
| rs5743618 | TLR1 | Chiang | 1 |
| rs1800629 | TNFA | Ho-Pun-Cheung | 1 |
| rs6497759 | TNRC6A | Dreussi | 1 |
| rs139911 | TNRC6B | Dreussi | 1 |
| rs1642785 | TP53 | Dreussi | 1 |
| rs1042522 | TP53 | Ho-Pun-Cheung | 1 |
| rs2602141 | TP53 | Ho-Pun-Cheung | 1 |
| rs560191 | TP53 | Ho-Pun-Cheung | 1 |
| rs2273953 | TP73 | Ho-Pun-Cheung | 1 |
| rs1801173 | TP73 | Ho-Pun-Cheung | 1 |
| rs6088619 | TRBP | Dreussi | 1 |
| rs2853542 | TYMS | Balboa,Hur,Lamas,Paez,Sebio,Stoehlmacher | 6 |
| rs2847153 | TYMS | Boige | 1 |
| rs2298582 | TYMS | Boige | 1 |
| rs2612101 | TYMS | Boige | 1 |
| rs10502290 | TYMS | Boige | 1 |
| rs2260821 | TYMS | Boige | 1 |
| rs3744962 | TYMS | Boige | 1 |
| rs1001761 | TYMS | Boige | 1 |
| rs2853741 | TYMS | Boige | 1 |
| VNTR/5'UTR, | TYMS | Paez | 1 |
| rs1801019 | UMPS | Kim | 1 |
| rs2274507 | USP20 | Kim | 1 |
| rs2010963 | VEGF | Dreussi,Ho-Pun-Cheung | 2 |
| rs1570360 | VEGF | Dreussi,Ho-Pun-Cheung | 2 |
| rs3025039 | VEGF | Ho-Pun-Cheung,Hu-Lieskovan | 2 |
| rs699947 | VEGF | Ho-Pun-Cheung | 1 |
| rs2773354 | XPA | Boige | 1 |
| rs1800975 | XPA | Boige | 1 |
| rs2808667 | XPA | Boige | 1 |
| rs3176689 | XPA | Boige | 1 |
| rs3176757 | XPA | Boige | 1 |
| rs3176639 | XPA | Boige | 1 |
| rs3176683 | XPA | Boige | 1 |
| rs3176658 | XPA | Boige,Ho-Pun-Cheung | 2 |
| rs2805835 | XPA | Boige | 1 |
| rs25487 | XRCC1 | Balboa,Cecchin,Dreussi,Grimminger,Ho-Pun-Cheung,Lamas,Nicosia,Paez,Sebio,Formica | 10 |
| rs2293036 | XRCC1 | Boige | 1 |
| rs304729 | XRCC1 | Boige | 1 |
| rs2023614 | XRCC1 | Boige | 1 |
| rs3213255 | XRCC1 | Boige | 1 |
| rs3213266 | XRCC1 | Boige | 1 |
| rs3213334 | XRCC1 | Boige | 1 |
| rs2854496 | XRCC1 | Boige | 1 |
| rs1001581 | XRCC1 | Boige | 1 |
| rs1799782 | XRCC1 | Cecchin,Grimminger,Ho-Pun-Cheung,Paez | 4 |
| rs3213239 | XRCC1 | Dreussi | 1 |
| rs25489 | XRCC1 | Dreussi,Ho-Pun-Cheung,Paez | 3 |
| rs861539 | XRCC1 | Grimminger | 1 |
| rs3213245 | XRCC1 | Ho-Pun-Cheung | 1 |
| rs3212102 | XRCC3 | Boige | 1 |
| rs861531 | XRCC3 | Boige | 1 |
| rs861528 | XRCC3 | Boige | 1 |
| rs3212090 | XRCC3 | Boige | 1 |
| rs861530 | XRCC3 | Boige | 1 |
| rs3212079 | XRCC3 | Boige | 1 |
| rs12432907 | XRCC3 | Boige | 1 |
| rs1799794 | XRCC3 | Cecchin,Dreussi,Ho-Pun-Cheung | 3 |
| rs861539 | XRCC3 | Cecchin,Dreussi,Ho-Pun-Cheung,Hu-Lieskovan | 4 |
| rs1799796 | XRCC3 | Dreussi | 1 |
| rs1051677 | XRCC5 | Ho-Pun-Cheung | 1 |
| rs1051685 | XRCC5 | Ho-Pun-Cheung | 1 |
| rs2440 | XRCC5 | Ho-Pun-Cheung | 1 |
| rs6941 | XRCC5 | Ho-Pun-Cheung | 1 |
| rs4244146 | ZNF281 | Kim | 1 |
| rs2278415 | ZNF350 | Ho-Pun-Cheung | 1 |
| rs2278420 | ZNF350 | Ho-Pun-Cheung | 1 |
